# Cryptosporidial diarrhea in an immunocompetent adult: role of nitazoxanide

**DOI:** 10.3402/jchimp.v3i3-4.21075

**Published:** 2013-12-17

**Authors:** Naba Raj Mainali, Patrick Quinlan, Anene Ukaigwe, Shailaja Amirishetty

**Affiliations:** Department of Medicine, Reading Health System, West Reading, PA, USA

**Keywords:** Cryptosporidium, modified acid fast staining, nitazoxanide

## Abstract

Diarrhea caused by Cryptosporidium is most commonly seen in patients with HIV or AIDS or other immunocompromised conditions like diabetes mellitus, or patients on a high dose of steroids or immunosuppressants. The organism is a parasite that infects gastrointestinal epithelium, producing potential life-threatening diarrhea in people with AIDS but usually self-limiting diarrhea in immunocompetent hosts. Here in, we present an interesting case of persistent diarrhea caused by Cryptosporidium in an immunocompetent adult requiring treatment with nitazoxanide.

## Introduction

Cryptosporidiosis is commonly seen in patients with AIDS and other immunosuppressed conditions. It may also be seen in immunocompetent patients who might be asymptomatic or may present with acute or persistent diarrhea with abdominal pain and weight loss. It accounts for up to 6% of diarrhea in immunocompetent patients (1). A few numbers of *Cryptosporidium parvum* oocysts can contaminate even treated drinking water as they are extremely resistant to chemical disinfection and filtration (2). Cryptosporidium is an intracellular parasite that is transmitted by the ingestion of Cryptosporidium oocysts through contaminated water and food (2, 3). Among many species including *Cryptosporidium hominis*, *C. canis*, *C. suis*, *C. parvum* is the most common species that can infect human cells (4). One Cryptosporidium oocyst contains four sporozoites, each of which can infect intestinal epithelial cells. It is usually diagnosed with the detection of oocysts by modified acid-fast (MAF) staining of the organism on microscopical examination of the stool.

## Case report

A 43-year-old Caucasian woman with no significant past medical history presented to the emergency department complaining of diarrhea, abdominal discomfort, and nausea for the last 10 days. Her symptoms began with diarrhea, vomiting, nausea, and low-grade fever and after 3 days, her fever and vomiting resolved. However, she continued to have diarrhea and nausea. She went to one of the local medical centers and was given the provisional diagnosis of viral gastroenteritis. After a brief course of antiemetics and IV fluids, she was sent home. She had minimal relief and continued to have similar symptoms. On the 10th day since her symptom onset, she decided to come to our hospital for further evaluation. She denied fever, chills, abdominal pain, and burning micturition at the time of presentation. She is a lifetime non-smoker, non-alcoholic, and denied any history of allergy, unusual food intake, sick contacts and travel to other countries. She reported a loss of appetite and weight loss of 5 kg during this period. She denied any bloody stools. She gave no history of diabetes mellitus and HIV risk behavior.

Physical examination revealed blood pressure of 90/68 mm Hg and heart rate of 88/min with slightly dry oral mucosa. There was no pallor, icterus, lymphadenopathy, or rashes. Abdominal examination showed mildly tender abdomen with hyperactive bowel sounds. Heart, lungs, and neurological examination were essentially normal.

She was admitted with the probable diagnosis of viral diarrhea with the main differentials including infectious colitis, *Clostridium difficile* diarrhea or diarrhea associated with underlying colonic malignancy.

Laboratory findings revealed WBC count of 9,000/µL, platelets 260,000/µL, hemoglobin 13 gm/dL, sodium 141 meq/L, potassium 3.1 meq/L, magnesium 1.3 meq/dL, bicarbonate 28 meq/L, blood urea nitrogen (BUN) 7 meq/L, creatinine 0.75 mg/dL, blood sugar 88 mg/dL, bilirubin 0.6 mg/dL, AST 33 U/L, ALT 39 U/L, and lipase 25 U/L. CT scan of the abdomen and pelvis revealed gas and fluid distended colon without pericolonic strandings and colonic wall thickening. Colonoscopy showed only mild proctitis. Rota virus antigen, Giardia antigen, *C. difficile* toxin, HIV 1 and 2 antibodies were all negative. Cryptosporidium oocysts were detected by positive MAF staining of the organism on stool. Also, a stool test for Cryptosporidium antigen was found to be positive.

She was given normal saline, electrolyte replacement including magnesium and potassium. She received nitazoxanide 500 mg per oral twice daily for a total of 3 days as per infectious disease consultation. She slowly recovered with less frequent bowel movements without any obvious side effects from the medication and became completely symptom free on the fifth day of starting treatment.

She was then discharged without any anti-parasitic agents. She did not have recurrence of her diarrhea on her 1-month follow-up visit with her primary care physician.

## Discussion

Cryptosporidium is associated with persistent, potential life-threatening diarrhea in AIDS patients or patients with other immunocompromised conditions. *C. parvum* is a unique organism as it lacks host and organ specificity. It also has resistance to many antimicrobial agents and an ability for autoinfection (5). Since 1983, along with the evolution of the AIDS epidemic, Cryptosporidium emerged as a life-threatening infection in this particular subpopulation (6–8). People with AIDS most commonly suffer from more severe and prolonged diarrhea that can lead to a fatal outcome.

In immunocompetent hosts, Cryptosporidium can cause acute or persistent diarrhea associated with low-grade fever, nausea, vomiting, and weight loss. Children aged less than 1 year are especially susceptible. Symptoms in adults are usually limited to the gastrointestinal tract (1).

The diagnosis of Cryptosporidiosis should be considered in all immunocompromised patients who present with acute or persistent diarrhea (1). Confirmatory diagnosis of cryptosporidiosis requires microscopical detection of the parasite in tissues or body fluids (1, 9). The easiest and quickest method of detecting oocysts is MAF staining of the organism on microscopical examination of the stool (1, 8, 9).

Cryptosporidiosis in immunocompetent hosts is usually self-limiting and no specific treatment is required unlike AIDS patients who require highly active anti-retroviral treatment (HAART) and/or antiparasitic agents (1, 9). It is associated with high mortality if it affects infants and young children in developing countries (1). Although there is no specific antimicrobial therapy proven to eradicate Cryptosporidium, there are some agents to suppress the infection (1, 5, 10). Since the organism is intracellular, antimicrobial agents must first enter the host cell to effectively inactivate it or kill the host cells containing the intracellular organisms (9). Nitazoxanide is a thiazolide antimicrobial that reduces the load of parasites and is currently a first-in-class treatment for persistent, severe Cryptosporidiosis in immunocompetent individuals (11–16).
